# A Placebo-Controlled Exploratory Trial of Sirolimus for Tocilizumab-Resistant Idiopathic Multicentric Castleman Disease: Early Termination and Long-Term Extension Results Based on Descriptive Results From Two Patients

**DOI:** 10.7759/cureus.98233

**Published:** 2025-12-01

**Authors:** Tomohiro Koga, Remi Sumiyoshi, Toshimasa Shimizu, Naoki Hosogaya, Chizu Fukushima, Hiroshi Yamamoto, Hajime Yoshifuji, Shinji Higa, Atsushi Kawakami

**Affiliations:** 1 Department of Immunology and Rheumatology, Division of Advanced Preventive Medical Sciences, Nagasaki University Graduate School of Biomedical Sciences, Nagasaki, JPN; 2 Clinical Research Center, Nagasaki University Hospital, Nagasaki, JPN; 3 Department of Rheumatology and Clinical Immunology, Kyoto University, Kyoto, JPN; 4 Department of Internal Medicine, Division of Rheumatology, Daini Osaka Police Hospital, Osaka, JPN

**Keywords:** chap score, idiopathic multicentric castleman disease, mtor inhibitor, randomized controlled trial, sirolimus

## Abstract

Background

Idiopathic multicentric Castleman disease (iMCD) remains challenging to treat, with a considerable number of patients showing insufficient response to tocilizumab, the current standard therapy for iMCD. Sirolimus, an mTOR inhibitor, is a potential therapeutic option for tocilizumab-resistant cases based on its mechanism of action and preliminary case reports.

Methods

This investigator-initiated, multicenter, exploratory, placebo-controlled study was designed to evaluate sirolimus (2 mg daily) versus placebo in patients with tocilizumab-resistant iMCD. The primary endpoint was the change in the CHAP (CRP, Hemoglobin, Albumin, Performance Status) score from baseline at week 16. The study was prematurely terminated due to recruitment challenges and the impact of the COVID-19 pandemic, enrolling only two of the planned 20 participants. Both patients completed a 40-week open-label extension phase of sirolimus treatment.

Results

Two patients were randomized (one sirolimus and one placebo). The primary endpoint of the CHAP score reduction was not achieved during the 16-week double-blind phase. However, the sirolimus-treated patient maintained disease stability (CHAP score remained at 1), whereas the placebo patient experienced disease progression (CHAP score increased from 3 to 5). The secondary outcomes showed contrasting patterns: the sirolimus patient demonstrated improvements in the physician’s global assessment and quality of life measures, while the placebo patient showed deterioration. During the extension phase, the patient initially assigned to placebo experienced significant improvement after switching to sirolimus (CHAP score decreased from 5 to 3, meeting the criteria for a clinically meaningful response). No serious adverse events were reported during the 40-week study period.

Conclusions

While the study's early termination with only two patients prevents definitive efficacy conclusions, the contrasting disease trajectories between treatment groups and the improvement observed when the placebo patient switched to sirolimus suggest a potential therapeutic benefit. The favorable safety profile of extended sirolimus treatment supports further investigation in larger, adequately powered studies of tocilizumab-resistant iMCD.

## Introduction

Idiopathic multicentric Castleman disease (iMCD) is a rare, life-threatening lymphoproliferative disorder characterized by systemic inflammation, constitutional symptoms, and dysregulated cytokine production, particularly interleukin-6 (IL-6) [1-4]. The estimated incidence ranges from 1,000 to 5,000 new cases annually in the United States, making it an orphan disease with limited treatment options [4]. The humanized anti-IL-6 receptor monoclonal antibody tocilizumab has emerged as a cornerstone therapy for iMCD, demonstrating efficacy in multiple studies and receiving regulatory approval [5]. However, clinical experience has revealed that approximately 40% of patients exhibit insufficient response to tocilizumab, creating a significant unmet medical need for alternative therapeutic approaches [5].

Recent research has identified dysregulation of the PI3K/AKT/mTOR signaling pathway in iMCD pathogenesis, particularly in IL-6 blockade-refractory cases [6]. The mammalian target of rapamycin (mTOR) is a central regulator of cell growth, metabolism, and immune function, making it an attractive therapeutic target [7]. Sirolimus (rapamycin), an FDA-approved mTOR inhibitor with an established safety profile in transplant medicine [8] and other autoimmune conditions, has shown promising results in case reports of tocilizumab-resistant iMCD [9-11].

This exploratory, placebo-controlled study was designed to provide the first randomized evidence regarding the efficacy and safety of sirolimus in patients with tocilizumab-resistant iMCD. While recruitment challenges led to early termination with a sample size smaller than planned, the study offers valuable insights into the potential role of mTOR inhibition in this difficult-to-treat population and provides important safety data for extended sirolimus use in patients with iMCD.

## Materials and methods

Study design and setting

This investigator-initiated, multicenter, exploratory, placebo-controlled study with an open-label long-term extension (LTE) was conducted at eight medical centers across Japan. This study was registered in the Japan Registry of Clinical Trials (jRCT2071190029 and jRCT2051200050). The original protocol and amendments were approved by the Institutional Review Board of each participating center. The trials were conducted in accordance with the SPIRIT guidelines. The protocols for these trials (NUH03iMCD and NUH04iMCD) have been published previously [12,13].

Participants

Recruitment was conducted between October 2019 and April 2021. Two patients diagnosed with tocilizumab-resistant iMCD were enrolled in this study, although the target enrollment was 20. The inclusion criteria were a definitive diagnosis of iMCD based on the Japanese diagnostic criteria and failure to achieve complete remission with tocilizumab treatment for more than eight weeks. Patients were required to be ≥ 18 years of age and maintain a stable dose of corticosteroids prior to the start of the study. The exclusion criteria were designed to eliminate patients with a high risk of adverse outcomes due to drug or disease-related complications.

Randomization and blinding

The participants were randomly assigned in a 1:1 ratio to receive sirolimus or placebo. Randomization was implemented using a biased coin design with an imbalanced tolerance to maintain allocation concealment and treatment balance. The initial phase maintained a double-blind structure, with both the participants and investigators being unaware of the treatment assignments.

Interventions

In the controlled trial phase, the assigned medication was administered orally at a daily dose, with the sirolimus group receiving 2 mg of sirolimus once daily for 16 weeks. The extension phase involved open-label administration of sirolimus, in which both patients, regardless of their initial assignment, were administered sirolimus under the same dosing schedule.

Outcomes

The primary endpoint was the change in the CHAP (CRP, Hemoglobin, Albumin, Performance Status) score from the baseline to week 16. The CHAP score is a validated disease activity assessment tool specifically developed for iMCD by Fujimoto et al. [5]. The score integrates four key disease parameters: CRP (C-reactive protein), Hemoglobin, Albumin, and Performance Status (ECOG), each graded on a five-point scale (0-4). The total score, therefore, ranges from 0 to 16. Secondary outcomes included hemoglobin, albumin, and CRP levels; changes in lymph node size; patient and physician global assessment; and safety parameters, including adverse events. In the extension phase, these outcomes were monitored to assess the long-term efficacy and safety of the treatment. Secondary outcomes included hemoglobin, albumin, and CRP levels; changes in lymph node size; patient and physician global assessment; and safety parameters, including adverse events. In the extension phase, these outcomes were monitored to assess the long-term efficacy and safety of the treatment.

Sample size and statistical considerations

The study was originally designed to enroll 20 participants (10 per group) based on feasibility considerations, given the rarity of tocilizumab-resistant iMCD. This sample size was determined through consultation with iMCD experts and consideration of patient availability across the participating centers, rather than formal power calculations. This study was designed as a proof-of-concept trial to generate preliminary efficacy and safety data to inform future larger studies.

Early termination and modified analysis plan

The trial was terminated early after enrolling only two participants due to (1) unexpected difficulties in obtaining matching placebo supplies when the manufacturer changed the sirolimus formulation and (2) recruitment challenges exacerbated by the COVID-19 pandemic, which significantly reduced patient willingness to participate in clinical trials requiring frequent hospital visits. Given the extremely small sample size, formal statistical hypothesis testing was not conducted. Instead, descriptive analyses were conducted, and the results are presented as individual patient trajectories and summary statistics. The analysis focused on documenting safety outcomes and exploring preliminary efficacy signals to inform future trial designs.

Safety monitoring

Adverse events were monitored and recorded throughout the trial and extension phases. All events were categorized according to their severity and relationship with the study drug. Clinical examinations, laboratory assessments, and other medically important indicators were systematically documented.

Data collection and management

Clinical data were collected and managed using an electronic data capture system compliant with Japan's Good Clinical Practice guidelines. Patient confidentiality was strictly maintained throughout the study, with anonymized and securely stored data being used.

Ethical considerations

The study protocol was approved by the institutional review boards of each site, and all participants provided written informed consent prior to participation. This study was conducted in accordance with the Declaration of Helsinki and other relevant ethical guidelines.

## Results

Participant enrollment and characteristics

**Figure 1:**
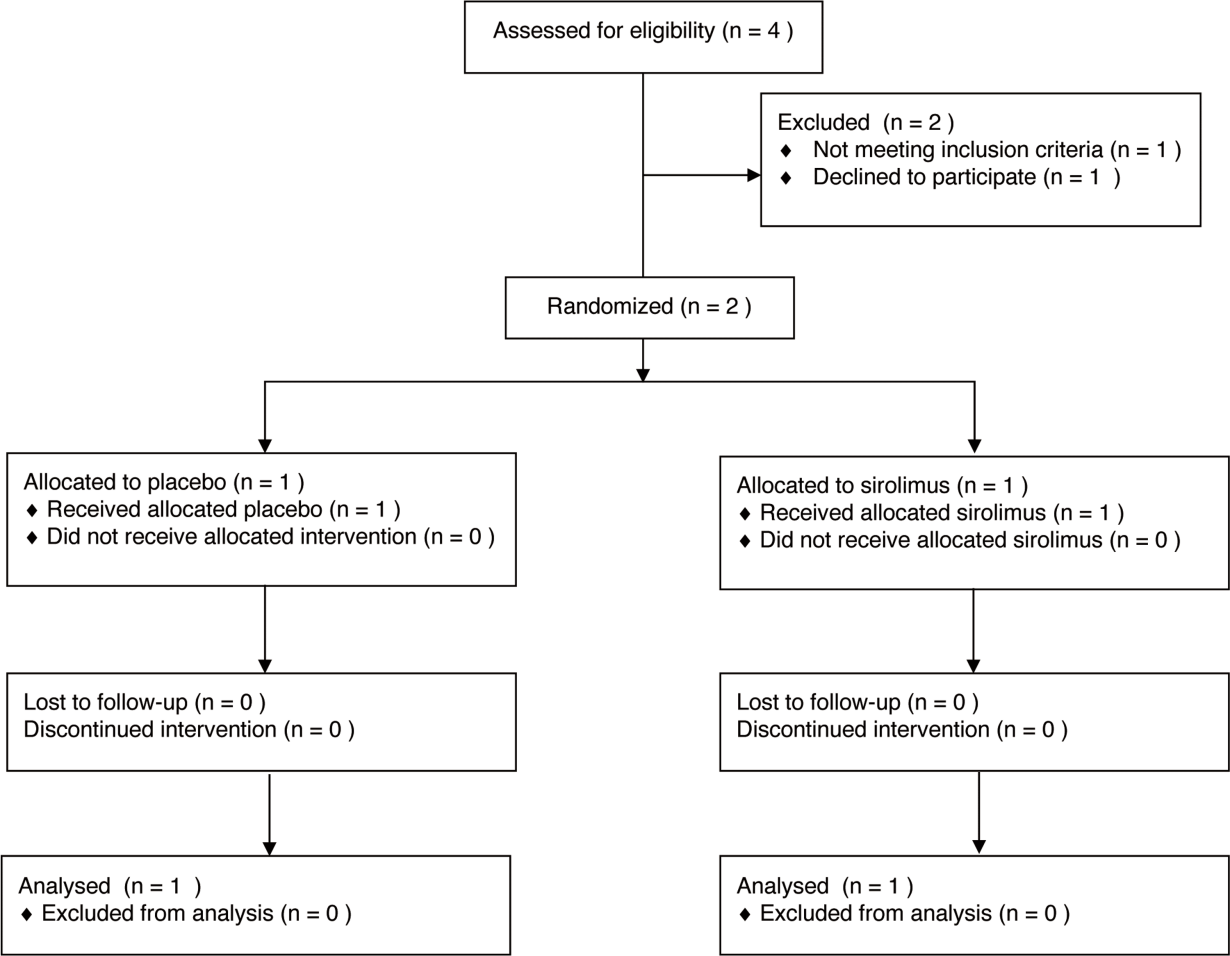
Participant flow diagram. This figure illustrates the flow of participants through a clinical trial. Starting with the initial screening and moving through subsequent phases of the trial, including enrollment, randomization, and follow-up, the diagram details the number of participants at each stage.

As shown in Figure 1, in the preliminary trial (NUH03iMCD), four cases of consent were obtained and screened, of which two patients were enrolled and one was allocated to the study drug group and one to the placebo group. In addition, two patients dropped out of the study due to withdrawal of consent from the patient and failure to meet the eligibility criteria. Two patients with tocilizumab-refractory iMCD were randomized 1:1 to receive either sirolimus or a placebo. Notably, the study was prematurely terminated because of unexpected difficulties in obtaining placebo supplies. These challenges were further compounded by the COVID-19 pandemic, which significantly hindered participant recruitment and retention, preventing us from reaching the target enrollment of 20 patients.

In the LTE trial (NUH04iMCD), participants who had completed the 16-week double-blind phase were included in a long-term safety and efficacy study of sirolimus. The study included one participant (C04-01 from the sirolimus group) at Kyoto University Hospital and another (C06-02 from the placebo group) at Daini Osaka Police Hospital. After the initial 16-week double-blind phase, the participant (C06-02), originally in the placebo group, began receiving sirolimus. Owing to the premature termination of the trial, all participants were eventually classified as discontinued subjects. The patient demographics and baseline characteristics are shown in Table 1.

**Table 1:** Baseline characteristics of study participants. The table summarizes the baseline demographic and clinical characteristics of the study participants. The table includes information on sex, age, race, pathological findings of the lymph nodes, Body Mass Index (BMI), C-Reactive Protein (CRP) levels, white blood cell count, hemoglobin levels, platelet count, albumin levels, creatinine levels, immunoglobulin G (IgG) levels, Eastern Cooperative Oncology Group Performance Status (ECOG PS), and Castleman's Disease Hybrid Assessment of Performance Status (CHAP) score.

	C04-01	C06-02
Gender	female	male
Age	47	61
Race	Asian	Asian
Pathological findings of lymph nodes	Plasma cell type	Hyaline vascular type
BMI (kg/m^2^)	19.6	32.3
CRP (mg/L)	19	43
White blood cells (/μL）	7900	9400
Hemoglobin (g/dL)	13.1	10.4
Platelet count (×10^3^/μL)	312	200
Albumin (g/dL)	4.3	3.2
Creatinine (mg/dL)	0.5	1.5
IgG (mg/dL)	1030	2210
ECOG PS	0	1
CHAP Score	1	

Treatment exposure and compliance

Both participants completed the full 16-week double-blind phase with excellent medication adherence (99.2% for C04-01, 100% for C06-02). Subsequently, both enrolled in the extension phase receiving open-label sirolimus for an additional 24-40 weeks, with total treatment durations of 413 days (C04-01) and 420 days (C06-02). Sirolimus blood levels confirmed adequate drug exposure throughout both study phases (Table 2).

**Table 2:** Sirolimus blood concentration measurements during the double-blind phase. The table presents the sirolimus blood concentration measurements for the two participants over the course of the trial. Sirolimus concentrations in the sirolimus-treated participant (C04-01) indicate therapeutic levels, while negligible or non-detectable levels in the placebo participant (C06-02) underscore the double-blind administration phase.

Group	Subject Number	Visit	Sirolimus Blood Concentration (ng/mL)
Sirolimus	C04-01	1 Week	9.2
		2 Weeks	13.5
		4 Weeks	12.5
		8 Weeks	11.6
		12 Weeks	23.2
		16 Weeks	27.8
Placebo	C06-02	1 Week	1.0 (Not detected)
		2 Weeks	1.0 (Not detected)
		4 Weeks	1.0 (Not detected)
		8 Weeks	1.0 (Not detected)
		12 Weeks	1.0 (Not detected)
		16 Weeks	1.0 (Not detected)

Primary outcome in the 16-week, double-blind phase (NUH03iMCD trial)

Table 3 shows the longitudinal clinical and laboratory data of the two participants enrolled in the NUH03iMCD trial. The primary efficacy endpoint, defined as a decrease of ≥1 in the CHAP score from baseline to 16 weeks, was not observed in either participant. Specifically, the sirolimus-treated patient (C04-01) had a stable CHAP score of 1 at both the baseline and week 16, indicating no change. In contrast, the placebo-treated patient (C06-02) experienced an increase in the CHAP score from 3 at baseline to 5 at week 16, suggesting disease progression rather than improvement. These results did not meet the predefined criterion for efficacy based on the CHAP score reduction within the 16-week treatment period.

**Table 3:** Longitudinal clinical and laboratory data from the NHU03iMCD trials The table displays the longitudinal clinical and laboratory data of the two participants enrolled in the NUH03iMCD trial that assessed sirolimus versus placebo. The table lists CHAP scores and C-reactive protein (CRP), hemoglobin, and albumin levels at each visit from baseline up to the 12-month follow-up. The ECOG Performance Status (PS) scores were also provided to depict general patient well-being throughout the trial duration. *CRP Subscore is subtracted CRP from the CHAP Score

			CHAP Score		CRP	Hemoglobin	Albumin	ECOG PS
Group	Subject	Visit	CHAP Score	CRP Subscore*	(mg/dL)	(g/dL)	(g/dL)	PS
	Number							
Sirolimus	C04-01	Baseline	1	0	1.9	13.1	4.3	0
		2 Weeks	1	0	1.6	13.2	4.1	0
		4 Weeks	1	0	1.8	13.6	4.2	0
		8 Weeks	1	0	1.3	12.6	4.2	0
		12 Weeks	2	1	1.2	11.6	4.2	0
		16 Weeks	1	0	1.3	12.4	4.3	0
Placebo	C06-02	Baseline	3	2	4.3	10.4	3.2	1
		2 Weeks	5	4	3.3	8.7	2.9	1
		4 Weeks	5	4	4.5	8.6	2.9	1
		8 Weeks	5	4	2.4	8.5	2.7	1
		12 Weeks	6	5	4	7.9	2.6	1
		16 Weeks	5	4	4.6	8.5	2.8	1

Secondary outcomes

The secondary endpoints were laboratory markers and overall well-being. At week 16, neither group showed a reduction in hemoglobin (Hb), albumin (Alb), or C-reactive protein (CRP) levels from baseline. The physician's general assessment of disease activity showed a decrease in the sirolimus-treated patients and an increase in the placebo group, indicating an improvement and worsening of perceived disease activity, respectively. However, the patient self-assessment did not show any decrease in disease activity in either group (Table 4).

**Table 4:** Longitudinal visual analog scale scores data from the NHU03iMCD trials The table presents the visual analog scale (VAS) scores for disease activity as assessed by both physicians and patients across multiple visits during the study. The scores range from 0 to 100, with higher scores indicating greater disease activity or symptom severity.

	Subject		Physician	Patient
Group	Number	Visit	VAS	VAS
Sirolimus	C04-01	Baseline	16	0
		2 Weeks	10	47
		4 Weeks	27	52
		8 Weeks	28	49
		12 Weeks	27	49
		16 Weeks	11	50
Placebo	C06-02	Baseline	22	22
		2 Weeks	41	36
		4 Weeks	38	26
		8 Weeks	26	27
		12 Weeks	40	43
		16Weeks	40	38

Regarding the SF-36 quality of life scores, sirolimus-treated patients showed an increase in the bodily pain (BP), general health (GH), emotional role (RE), and mental health (MH) subscales, suggesting an improved quality of life. The physical functioning (PF) and social functioning (SF) subscales remained unchanged. The placebo group experienced a decrease in all SF-36 subscale scores, implying a decline in quality of life (Table 5).

**Table 5:** Longitudinal the SF-36 subscale scores data from the NHU03iMCD trials The table shows the SF-36 subscale scores of patients C04-01 and C06-02 over the course of the preliminary trial. Scores are provided for both the 1-100 score scale and norm-based (NB) scoring, where higher scores indicate better health status across the domains of Physical Functioning (PF), Role Physical (RP), Bodily Pain (BP), General Health (GH), Vitality (VT), Social Functioning (SF), Role Emotional (RE), and Mental Health (MH). The table tracks the evolution of quality of life measures from baseline to 16 weeks, reflecting changes under the treatment regimen with sirolimus versus placebo and highlighting improvements, declines, or stability in health perception over time.

	Subject		1-100 Score									NB Score								
																				Group	Number	Visit	PF	RP	BP	GH	VT	SF	RE	MH		PF	RP	BP	GH	VT	SF	RE	MH	
Sirolimus	C04-01	Baseline	85	100	62	37	43.8	100	91.7	55		47	55.7	44.7	36.2	40.2	57	51.9	41.1	
		4 Weeks	85	93.8	62	42	50	100	100	65		47	52.4	44.7	38.9	43.4	57	56.1	46.5	
		8 Weeks	85	93.8	100	42	43.8	100	100	75		47	52.4	61.7	38.9	40.2	57	56.1	51.8	
		12 Weeks	80	62.5	72	42	37.5	100	100	65		43.4	35.8	49.2	38.9	37	57	56.1	46.5	
		16 Weeks	85	75	100	42	37.5	100	100	75		47	42.4	61.7	38.9	37	57	56.1	51.8	
Placebo	C06-02	Baseline	80	93.8	72	52	50	100	100	70		43.4	52.4	49.2	44.2	43.4	57	56.1	49.1	
		4 Weeks	65	68.8	74	35	56.3	87.5	75	65		32.6	39.1	50.1	35.1	46.6	50.6	43.6	46.5	
		8 Weeks	70	81.3	62	35	50	75	75	65		36.2	45.8	44.7	35.1	43.4	44.1	43.6	46.5	
		12 Weeks	55	62.5	52	40	56.3	87.5	83.3	65		25.4	35.8	40.3	37.8	46.6	50.6	47.7	46.5	
		16 Weeks	55	68.8	52	40	43.8	75	66.7	60		25.4	39.1	40.3	37.8	40.2	44.1	39.4	43.8	

Throughout the study period, including the 16-week double-blind phase and the long-term extension phase (up to 420 days), systematic clinical and radiological assessments revealed no measurable lymph node enlargement or organomegaly in either participant. Neither participant achieved a complete response (CR) or partial response (PR) at week 16 based on the Castleman Disease Collaborative Network (CDCN) treatment response criteria. The CHAP score and the CHAP score minus the CRP score did not decrease from baseline for either participant.

**Figure 2:**
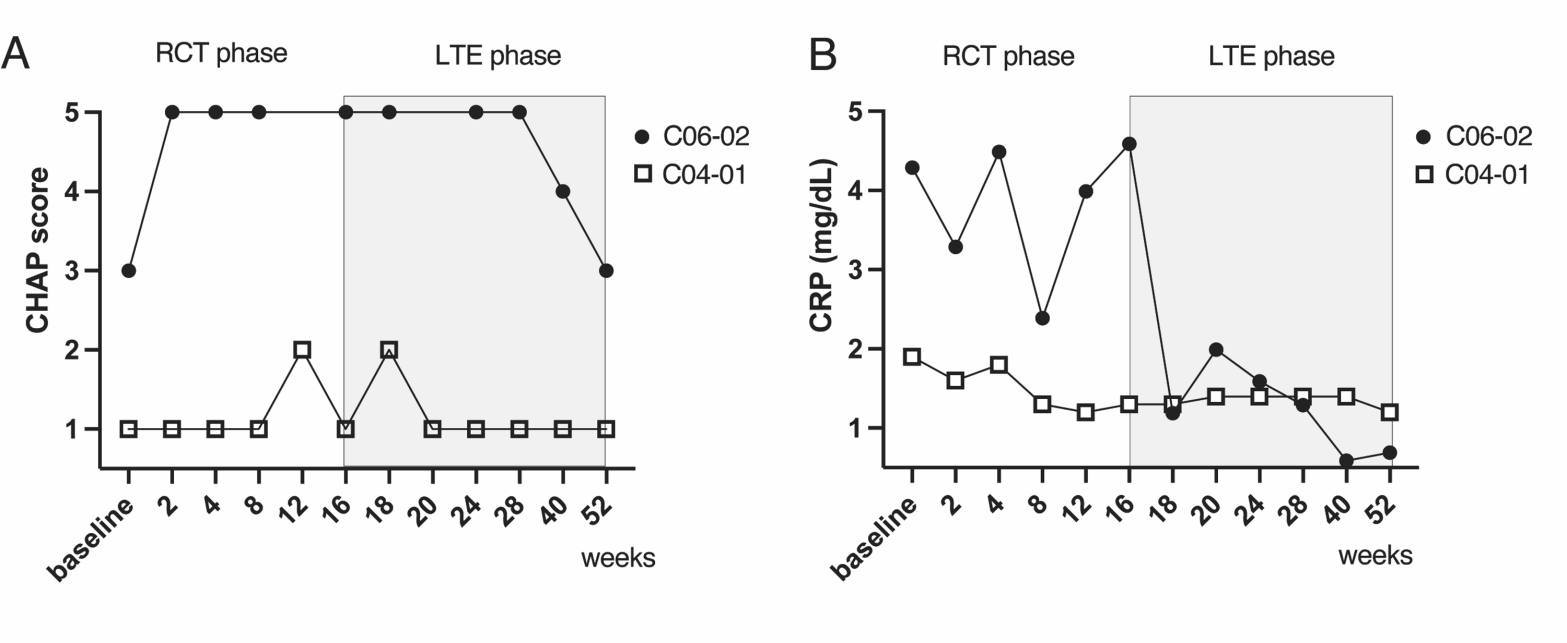
Longitudinal CHAP scores and CRP levels during RCT and LTE phases for participants C04-01 and C06-02. (A) CHAP score trajectories for participants C04-01 (represented by squares) and C06-02 (represented by circles) across the randomized controlled trial (RCT) phase and long-term extension (LTE) phase of the study. (B) CRP level trajectories for the same participants over the same period, showing the inflammatory response along with treatment administration.

Figure 2 illustrates the changes in CHAP scores and CRP levels throughout the course of the study, dividing the observations into RCT and LTE phases. As shown in Figure 2A, the CHAP scores for participant C04-01 remained stable and low throughout both the phases, indicating consistent disease control. In contrast, the CHAP scores in participant C06-02, which were initially high during the RCT phase, showed a significant decrease after transitioning into the LTE phase, reflecting an improvement in disease symptoms with extended treatment. Figure 2B depicts the CRP levels, where participant C04-01 maintained low inflammatory markers throughout the study, whereas participant C06-02 exhibited fluctuating but overall decreasing CRP levels in the LTE phases, further correlating with the clinical improvement observed in the LTE phase.

Safety outcomes

No serious adverse events were reported during either study phase. Mild adverse events included aphthous ulcers (considered possibly related to sirolimus), oral herpes, gastroenteritis, vaccination-related symptoms, and minor musculoskeletal complaints. All events were Grade 1-2 in severity. Laboratory monitoring revealed no clinically significant abnormalities attributable to study medication. The extended treatment duration (up to 420 days) provided valuable long-term safety data supporting the tolerability of sirolimus in this patient population.

## Discussion

Recent studies have revealed that iMCD involves not only excessive IL-6 signaling but also aberrant activation of the PI3K/AKT/mTOR pathway, which regulates cell growth, metabolism, and immune responses [14]. In particular, mTORC1 and mTORC2 activation has been demonstrated in lymph nodes from patients with iMCD-TAFRO, providing a biological rationale for mTOR inhibition [15]. Sirolimus, an mTOR inhibitor, may exert therapeutic effects by suppressing abnormal lymphoproliferation and cytokine production, thereby stabilizing disease activity in refractory cases [16,17].

This exploratory study represents the first prospective evaluation of sirolimus in tocilizumab-resistant iMCD, despite premature termination with only two participants. While the extremely small sample size prevents definitive efficacy conclusions, the contrasting disease trajectories observed between treatment groups and the systematic improvement when the placebo patient switched to sirolimus provide meaningful insights into the potential therapeutic role of mTOR inhibition in this challenging patient population.

The lack of CHAP score reduction in the sirolimus-treated patient prompts a discussion on the interpretation of 'efficacy' in a complex disease like iMCD, where stabilization might be as clinically significant as reduction, particularly in a disease known for its debilitating flares and progressive deterioration [4,18]. Hence, the stable CHAP score observed might suggest a beneficial effect of sirolimus, which was not captured by the stringent criteria of the primary endpoint. Although primary efficacy based on the CHAP score was not observed, secondary outcomes provided mixed signals. Physician-assessed improvement in the sirolimus-treated patient and quality of life measures that improved or remained stable in certain domains contrasted with the worsening condition in the placebo-treated patient.

Moreover, the improvement observed in the CHAP score as well as the CRP level during open-label LTE in the patient initially assigned to the placebo group further supports the potential efficacy of sirolimus. These outcomes imply a positive trend for sirolimus in managing iMCD, suggesting a suppressive effect on disease activity over time.

The fact that sirolimus was well tolerated with no new safety concerns arising over the long-term treatment period is also encouraging. The adverse events observed were consistent with the known safety profile of sirolimus, and none led to treatment discontinuation, indicating that sirolimus could be a viable option for patients who have exhausted other treatment options.

There are several recognized types of Castleman’s disease, including the hyaline vascular type, plasma cell type [18,19], and the more recently described TAFRO syndrome, which includes thrombocytopenia, anasarca, fever, reticulin fibrosis, and organomegaly [20,21]. The current trial did not enroll patients with a severe phenotype that is often associated with TAFRO syndrome [22-24]. However, the effectiveness of sirolimus has been demonstrated not only in iMCD-TAFRO [16], but also in iMCD-NOS (not otherwise specified) [6], suggesting a broader therapeutic potential for this treatment. The results of this trial support the findings of the previous case reports.

Unfortunately, this trial was prematurely concluded after enrolling only two participants, largely because of difficulties in obtaining placebo supplies. The supplier had to change the formulation of the sirolimus tablets, making it impossible to produce matching placebo tablets. Additionally, the spread of the COVID-19 pandemic significantly contributed to the challenges faced, particularly influencing patient reluctance towards frequent hospital visits, which further complicated trial execution.

We acknowledge several important limitations: (1) the extremely small sample size precludes statistical analysis; (2) early termination may introduce selection bias; (3) baseline differences between participants limit comparative interpretation; (4) single-country recruitment may limit generalizability; and (5) the lack of formal interim analysis plan for early termination decisions.

## Conclusions

In conclusion, although definitive conclusions on the long-term safety and efficacy of sirolimus in patients with tocilizumab-insufficiently responsive iMCD cannot be drawn from this trial alone, there were indications of efficacy, as evidenced by partial improvements in the CHAP scores and physicians’ global assessments. Over a period exceeding one year, the continuous administration of sirolimus did not raise any new safety concerns, suggesting a favorable safety profile for long-term use. In addition, the historical data collected in this stringent trial environment could be instrumental in guiding future research and improving patient care strategies for iMCD.
